# Shrimp miR-1000 Functions in Antiviral Immunity by Simultaneously Triggering the Degradation of Two Viral mRNAs

**DOI:** 10.3389/fimmu.2018.02999

**Published:** 2018-12-18

**Authors:** Yi Gong, Chenyu Ju, Xiaobo Zhang

**Affiliations:** Laboratory for Marine Biology and Biotechnology of Qingdao National Laboratory for Marine Science and Technology, College of Life Sciences, Zhejiang University, Hangzhou, China

**Keywords:** shrimp miR-1000, white spot syndrome virus gene, mRNA degradation, virus-host interactions, *wsv191* and *wsv407*

## Abstract

MicroRNAs (miRNAs) function as crucial suppressors of gene expression via translational repression or direct mRNA degradation. However, the mechanism of multi-gene regulation by a host miRNA in antiviral immunity has not been extensively explored. In this study, the regulation of two white spot syndrome virus (WSSV) genes by its host (*Marsupenaeus japonicus* shrimp) miRNA (shrimp miR-1000) was characterized. The miRNA target gene prediction showed that only two virus genes (*wsv191* and *wsv407*) might be the targets of miR-1000. The results of insect cell transfection assays revealed that shrimp miR-1000 could target multiple virus genes (*wsv191* and *wsv407*). The mRNA degradation analysis and RNA FISH (fluorescence *in situ* hybridization) analysis indicated that miR-1000 triggered the mRNA degradation of target genes through 5′-3′ exonucleolytic digestion *in vivo* and thereby inhibited the virus infection in shrimp. The miRNA-mediated 5′-3′ exonucleolytic digestion of target mRNAs stopped near the 3′UTR (3′untranslated region) sequence complementary to the seed sequence of miR-1000. Therefore, our study provided novel insights into how a host miRNA targeted multiple viral genes and prevented host from virus infection.

## Introduction

It is well known that virus invasion of its host and the host immune response against the virus depend on virus-host interactions (1). During virus-host interaction, the regulation of gene expression plays an essential role. Given that microRNAs (miRNAs) function as crucial regulators of gene expression (2), their roles in virus-host interactions have attracted more attention in recent years. It has been reported that the expression levels of host miRNAs and virus miRNAs can be altered during virus infection (3–6), suggesting that miRNAs may exert great effects on virus-host interactions (7). The silencing of host *Drosha* and *Dicer*, two genes required for miRNA biogenesis, leads to a decreased mature miRNAs production and increases host sensitivity to virus infection (8). These findings suggest that host and virus miRNAs are required in the regulation of virus-host interactions. In shrimp, the host miR-7 can target the 3′untranslated region (3′UTR) of the WSSV (white spot syndrome virus) early gene *wsv477* to inhibit virus infection (9). Similarly, host miR-1000 regulates the apoptotic activity of shrimp and influences WSSV infection by targeting the shrimp *p53* gene (10). On the other hand, virus miRNAs can target host genes or virus genes to promote virus infection (11). In this context, the interactions between miRNAs and their target mRNAs play essential roles during virus infection. Since during virus infection, a large number of immune-related genes are strictly regulated by miRNAs, it would be of interest to explore how host miRNAs effectively regulate the expressions of target genes.

Typically, a mature miRNA, ~22 nucleotides (nt) in length, regulates its target gene expression through the binding of the miRNA seed sequence to the 3′UTR of mRNA, resulting in the translation repression or the direct mRNA degradation of target gene (12). The miRNA seed sequence may be complementary to the 3′UTRs of many mRNAs, thus a single miRNA may target multiple genes. It has been reported that the level of miR-155 is increased in CM758 cells during reticuloendotheliosis virus strain T (REV-T) infection, hence, promoting cell survival through targeting JARID2 (jumonji and AT-rich interaction domain containing 2) (13). Similarly, Epstein-Barr virus LMP1 (latent membrane protein-1) can induce the expression of miR-155, which further suppresses apoptosis by targeting the *Mcl-1* gene (BCL2 family apoptosis regulator) (14). It has also been shown that miR-155 can decrease the expression of C/EBP-b-β (CCAAT/enhancer-binding protein-β) in HepG2 cells during HBV (hepatitis B virus) infection (15). All these studies seem to suggest that an individual miRNA could target different genes in different cells. However, the mechanism of multi-gene regulation by a single miRNA during virus infection has not been extensively explored.

In this study, we sought to explore how a single miRNA could target and regulate multiple genes, by studying miR-1000, a host miRNA, which is upregulated during WSSV infection in *Marsupenaeus japonicus* shrimp. The white spot syndrome virus (WSSV), a member of the *Nimaviridae* family and *Whispovirths* genus, has become one of the most dangerous and devastating marine pathogens that affect crustaceans (6). As an enveloped rod-shaped virus, WSSV contains a double-stranded circular genomic DNA of about 300 kb, which has the capacity to encode 180 viral proteins and 89 viral miRNAs (6, 16). It has recently been reported that WSSV and shrimp are a good model for the investigation of virus-host interactions (17). The results from the present study have shown that miR-1000 played a negative role in WSSV infection, further data revealed that shrimp miR-1000 could simultaneously target two viral genes (*wsv191* and *wsv407*), which triggered mRNAs degradations through 5′-3′ exonucleolytic digestion, leading to the inhibition of virus infection. Therefore, the present data provides novel insights into how host miRNA targets multiple viral genes thus preventing host cells from the virus infection.

## Materials and Methods

### Shrimp Culture, Virus Infection, and Shrimp Mortality Analysis

Shrimp culture, virus infection and shrimp mortality analysis were conducted according to our previous study (3). Shrimp (*Marsupenaeus japonicus*) of ~15 g each were raised in tanks (60 cm × 40 cm × 30 cm) filled with aerated seawater at 20°C. The seawater salinity was 21–26%0. For each treatment, 20 individuals were used. To ensure that the shrimp were virus-free before experiments, three shrimp of each treatment were randomly selected and subjected to PCR with WSSV-specific primers (5′-TATTGTCTCTCCTGACGTAC-3′ and 5′-CACATTCTT CACGAGTCT AC-3′). At the same time, the shrimp were subjected to the detection of pathogenic microorganisms using a commercial kit for shrimp pathogen detection (Hangzhou Zhongce Bio-Sci & Tech Co. Ltd., Hangzhou, China), which could detect acute hepatopancreatic necrosis disease, yellow head virus, shrimp iridovirus, enterocytozoon hepatopenaei, covert mortality nodavirus, Taura syndrome virus, infectious hypodermol and hematopoietic necrosis virus, hepatopancreatic parvovirus, infectious myonecrosis virus, Vibrio cholera and Vibrio parahemolyticus. The detection indicated that the shrimp were pathogen-free. To infect shrimp with WSSV, 100 μl of WSSV (10^4^ copies/ml) prepared in sterile phosphate buffered saline (PBS) was intramuscularly injected into each virus-free shrimp at the lateral area of the fourth abdominal segment using a syringe with a 29-gauge needle. At different time points post-infection, shrimp were collected and stored in liquid nitrogen for later use. At the same time, the cumulative mortality of shrimp was examined daily. In shrimp mortality assays, shrimp without any treatment and shrimp injected with PBS were used as negative controls. The WSSV used was obtained as described previously (18). A small part of negative control shrimp died during shrimp mortality assays, because the laboratorial culture conditions for shrimp could not completely afford normal survival of shrimp.

Every experiment, including virus infection and shrimp mortality assay, was biologically repeated for three times. These data were used for statistical analysis.

### Northern Blot Analysis

Northern blotting was carried out to examine the expression levels of miRNA and mRNA (19). For the miRNA Northern blotting, miRNAs were extracted from shrimp hemocytes using the mirVana miRNA isolation kit (Ambion, USA) according to the manufacturer's instructions. On the other hand, for Northern blot analysis of mRNA, total RNAs were extracted with RNAprep Pure Cell/Bacteria Kit (Tiangen Biotech, China). After electrophoresis for 2 h, the RNAs were transferred onto a nylon membrane (Amersham Biosciences, UK), followed by UV cross-linking. The membrane was pre-hybridized in DIG (digoxigenin) Easy Hyb granule buffer (Roche, Switzerland) for 0.5 h at 42°C and then hybridized with DIG-labeled miR-1000 probe (5′-TACTGCTGTGACGGGACAATAT-3′), U6 probe (5′-GGGCCATGCTAA TCTTCTCTGTATCGTT-3′), wsv191 probe (5′-TTCTTGGCTGCAGTTGAAACCC AGCGAACCCT-3′), wsv407 probe (5′-CTCTCCACCCTTTCAATGATGGTAATGG AAGAAC-3′) or β-actin probe (5′-ATGTCACGAACGATTTCTCGCTCGGCGGTG-3′) at 42°C overnight. In shrimp, β-actin is used as an internal control (19). The detection was done with the DIG High Prime DNA labeling and detection starter kit II (Roche).

### The Silencing or Overexpression of miR-1000 in Shrimp

To silence or overexpress miR-1000 in shrimp, anti-miR-1000 oligonucleotide (AMO-miR-1000) or miR-1000 was delivered into shrimp by injection according to our previous study (19). AMO-miR-1000 (5′-ACTGCTGTGACGGGAC**A**AT**A**T-3′) and miR-1000 (5′-ATAT TGTCCCGTCACA**G**CA**G**T-3′) were modified with 2′-O-methyl (OME) (bold letters) and phosphorothioate (the remaining nucleotides). As controls, the sequences of miR-1000 and AMO-miR-1000 were randomly scrambled, generating miR-1000-scrambled (5′-AACTTGCGTCCGATAGTACCT-3′) and AMO-miR-1000- scrambled (5′-TAAGGGTAGTGAACCCAGCTT-3′), respectively. All oligonucleotides were synthesized by Sangon Biotech (Shanghai, China). AMO-miR-1000 or miR-1000, at a concentration of 15 μg/shrimp, was intramuscularly injected into shrimp at the lateral area of the fourth abdominal segment. At different time points after the last injection, shrimp hemocytes were collected for later use. The experiments silencing or overexpressing miR-1000 in shrimp were conducted independently for three times.

### Analysis of WSSV Copies Using Quantitative Real-Time PCR

Total genomic DNA was extracted from WSSV-infected shrimp with a DNA isolation kit (Omega, USA) according to the manufacturer's manual. The WSSV copies were then quantified using WSSV-specific primers (5′-TTGGTTTCATGCCC GAGATT-3′ and 5′-CCTTGGTCAGCCCCTTGA-3′) and TaqMan probe (5′-FAM- TGCTGCCGTCTCCAA-TAMRA-3′) according to previous studies (10, 19). A DNA fragment of 1,400 bp from the WSSV genome was used as the internal standard (20). The real-time PCR was conducted as described previously (10), with a total reaction volume of 25 μl comprising 12.5 μl of Premix Ex Taq (Takara, Japan), 0.25 μl of 10 μM primers each, 0.5 μl of 10 μM fluorogenic probe, and 100 ng of DNA template. For each treatment, quantitative real-time PCR was independently performed for three times to quantify WSSV copies.

### Prediction of Genes Targeted by miR-1000

In order to predict the viral genes targeted by miR-1000, two softwares including Targetscan (21) and miRanda (22) were used to predict the miR-1000-targeted sites in the 3′UTRs (untranslated regions) of WSSV genes. The target gene predicted was conducted by LC-Bio Technologies Co., Ltd. (Hangzhou, China). The software version of miRanda was miRanda v3.3a, and the operating system was ubuntu 16.4. The WSSV UTRs (untranslated regions) were collected from NCBI database (GenBank accession no AF440570.1) through perl script. The analysis parameters were as follows: –sc = 140 (the complementary matching score threshold of miRNA and target gene 3′UTR), –en = −15 kcal/mol (the complementary binding free energy threshold of miRNA and target gene 3′UTR), –scale = 4 (the match score weighted value of the first 11 bases of the miRNA). The software version of Targetscan was Targetscan7.0 and the operating system was ubuntu 16.4. The software command was targetscan_70.pl miRNA_file UTR_file Predicted Targets Output File. The UTR_file was obtained from NCBI database (GenBank accession no AF440570.1) through perl script. The muscle software was used for global comparison to get the alignment file, and then the alignment file was organized into the required format through perl script. Finally, the prediction data was presented in the Predicted Targets Output File after running the software.

### Plasmids Construction

To evaluate the interactions between miR-1000 and the genes *wsv191, wsv407* and *wsv024*, the *wsv191* 3′UTR, *wsv407* 3′UTR, and *wsv024* 3′UTR were cloned into the pIZ/V5-His vector (Invitrogen, USA) which carried the sequence of enhanced green fluorescent protein (EGFP) gene. As a control, the sequence of *wsv191* 3′UTR and *wsv407* 3′UTR matching the seed sequence of miR-1000 was mutated to yield the EGFP-Δ*wsv191*-3′UTR and EGFP-Δ*wsv407*-3′UTR construct. All the recombinant plasmids were confirmed by sequencing.

### Cell Culture, Transfection, And Fluorescence Assays

Cell culture, transfection and fluorescence assays were carried out as described previously (19). Insect High Five cells (Invitrogen, USA) were cultured in a 96-well plate with Express Five serum-free medium (SFM) (Invitrogen, USA) containing L-glutamine (Invitrogen, USA) at 27°C. The cells in each well were co-transfected with 0.2 μg of the constructed plasmids and 100 nM of miR-1000 or miR-1000-scrambled with Cellfectin II Reagent (Invitrogen, USA) according to the manufacturer's instructions. All the miRNAs were synthesized by Shanghai GenePharma (Shanghai, China). At 48 h after the co-transfection, the fluorescence intensity of cells was determined using a Flex Station II microplate reader (Molecular Devices, USA) at 490/510 nm excitation/emission (Ex/Em). Based on the fluorescence values of the cells co-transfected with miR-1000 and EGFP-wsv191-3′UTR, EGFP-wsv407-3′UTR or EGFP-wsv024-3′UTR, the relative fluorescence intensity was calculated. This assay was biologically repeated for three times.

### Quantification of mRNA Using Real-Time PCR

To determine the mRNA level of *wsv191* and *wsv407*, quantitative real-time PCR was performed with the wsv191-specific primers (5′-TTGACGAGGAGGATTGTAA AGG-3′ and 5′-ATACCAGGGTTTATTTTGTTGCG-3′) or wsv407-specific primers (5′-AACCCATTCCACCCCAATATC-3′ and 5′-ATATCTTTGTCGGCCAACTTGTC-3′) as described before (19). Shrimp β-actin was used as an internal control (primers 5′-CGAGCACGGCATCGTTACTA-3′ and 5′-TTGTAGAAAGTGTGATGCCAGAT CT-3′). Total RNAs were extracted from shrimp hemocytes using RNAprep Pure Cell/Bacteria kit (Tiangen Biotech, China). The cDNA was synthesized with PrimeScript^TM^ 1st strand cDNA synthesis kit (Takara, Japan). Quantitative real-time PCR reaction mixture contained 5 μl of SYBR® Premix Ex Taq, 0.5 μl of 10 μM forward and reverse primers and 100 ng of cDNA template. The PCR reaction conditions were: 95°C for 1 min, followed by 45 cycles of 30 s at 95°C, 30 s at 52°C, and 30 s at 72°C. For each treatment, quantitative real-time PCR was independently carried out for three times to quantify mRNAs.

### Western Blot Analysis

Western blot analysis was carried out according to our previous study (19). Shrimp hemocytes were collected and lysed with RIPA buffer (Beyotime Biotechnology, China) containing 2 mM phenylmethanesulfonyl fluoride (PMSF). After protein quantification with the enhanced BCA protein assay kit (Beyotime Biotechnology, China), the samples were subjected to electrophoresis (SDS-PAGE) for 45 min at 200 V. The proteins were then transferred onto a nitrocellulose membrane (Millipore, USA), followed by incubation of the membranes with blocking buffer [5% milk in TBST (Tris-buffered saline and Tween-20)] for 1 h at room temperature, and then with primary antibody (prepared in-house) at 4°C overnight. After washing 3 times with TBST, membranes were incubated with HRP (horseradish peroxidase)-conjugated secondary antibody (Bio-Rad, USA) for 2 h at 4°C. Subsequently, membranes were incubated with ECL substrate (Thermo Scientific, USA) and subjected to chemiluminiscence detection.

### RNA Interference (RNAi) Assay in Shrimp

RNAi was conducted according to the procedure described before (11, 19). To silence the expression of wsv191 and wsv407 in shrimp, the sequence-specific siRNAs targeting the *wsv191* or *wsv407* gene were designed, which were designated wsv191-siRNA (5′-CAGTGACGGTAAATTGTGTACCGTT-3′) and wsv407-siRNA (5′-GGGTGG AGAGTTAGAAAGTTGTACA-3′), respectively, and synthesized by the *in vitro* transcription T7 kit (TaKaRa, Japan) according to the manufacturer's instructions. To knock down the expression of virus genes, shrimp were infected with WSSV, followed by injection of 15 μg/shrimp synthesized siRNA at 48 h post-infection. The siRNAs were diluted into the 100 μl siRNA buffer (50 mM Tris-HCl, 100 mM NaCl, pH 7.5). Twelve hours later, shrimp were re-injected with the same amount of siRNA per shrimp. The duration of siRNA was 72 h in shrimp. At different time points after the last siRNA injection, three shrimps were randomly selected per each treatment group and stored in liquid nitrogen for later use. The RNAi assay was biologically repeated for three times.

### miR-1000-Mediated Degradation of Target Genes mRNA

The mRNA degradation analysis was conducted as described before in our laboratory (19). To determine the degradation of target mRNA mediated by miR-1000, different concentrations of miR-1000 (0, 10, 20, and 40 nM) or miR-1000-scrambled (0, 10, 20, and 40 nM) and the 3′UTR of miRNA-1000 target mRNA were incubated with shrimp Ago1 complex in incubation buffer (10 mM ATP and 2 mM GTP) at 30°C for various time points (0, 0.5, 1, and 2 h). The shrimp Ago1 complex was obtained by immunoprecipitation with shrimp Ago1-specific antibody as described previously in our laboratory (19). The shrimp Ago1 complex was specific as detected by Western blot (19). The 3′UTR of wsv191, wsv407, and wsv024 were cloned with specific primers (wsv191, 5′-GATCA CTAATACGACTCACTATAGGGAATACATAAT-3′ and 5′-TGGTTAGAAGAGAGT GACGATA-3′; wsv407, 5′-GATCACTAATACGACTCACTATAGGGATGCCTCAA A-3′ and 5′-AAGAACAATGAACGGCATTACTACCC-3′; wsv024, 5′-TCCAGTTC TAATACATCAAAATCCTGTTC-3′ and 5′-TCCGTAGATGACTAACACGGCTGG CTGAAAG-3′). The 3′UTR was synthesized using an *in vitro* T7 transcription kit (TaKaRa, Japan) according to the manufacturer's instructions. After incubation, the mixture was subjected to electrophoresis on 1% agarose gel at 120 V for 30 min. Next, the RNA was transferred onto Hybond-N+ nylon membrane. After ultraviolet cross-linking, membranes were pre-hybridized in DIG Easy Hyb granule buffer (Roche, USA) for 0.5 h at 42°C and then hybridized with the DIG-labeled probe (*wsv191*, 5′-TTCTTGGCTGCAGTTGAAACCCAGCGAACCCT-3′; *wsv407*, 5′-CT CTCCACCCTTTCAATGATGGTAATGGAAGAAC-3′; *wsv024*, 5′-GATTTATCAA GTACGAAAAGGATATTTTACTTGCTG-3′) at 42 °C overnight. The detection was done with the DIG High Prime DNA labeling and detection starter kit II (Roche).

### Sequencing of the Remaining mRNA 3′UTR After miR-1000-Mediated Degradation

The remaining 3′UTR of the target mRNA after miR-1000-mediated degradation was separated using 1% agarose gel electrophoresis (19). The remaining mRNA fragments were then recovered with Zymoclean Gel RNA Recovery Kit (Zymo Research, USA), followed by reverse transcription into single strand cDNA using PrimeScript™ II 1st Strand cDNA Synthesis Kit with random 6 primers (TaKaRa, Japan). Next, the single strand cDNAs were processed into double strand cDNAs with Second Strand cDNA Synthesis Kit (Beyotime Biotechnology, China). The double strand cDNAs were then cloned into the pEASY®-Blunt Simple Cloning Vector (Transgen Biotech, China). The constructed DNA was ascertained by sequencing using the M13 universal primers.

### Fluorescence *in situ* Hybridization

Fluorescence *in situ* hybridization assay was carried out according to our previous study (19). Shrimp hemocytes were planted on a polylysine-coated glass slides and fixed with 4% polyformaldehyde for 15 min at room temperature. Slides were then dehydrated in 70% ethanol overnight at 4°C, followed by incubation with hybridization buffer [1 × SSC (15 mM sodium citrate, 150 mM sodium chloride, pH 7.5), 10% (w/v) dextran sulfate, 25% (w/v) formamide, 1 × Denhardt's solution] containing 100 nM probe for 5 h at 37°C. The probes used were miR-1000 probe (5′-FAM-TACTGCTGTGACGGGACAATAT-3′), wsv191 probe (5′-Cy3-ATCACCA GTGTTTCGTCATGGA-3′), wsv407 probe (5′-Cy5-AGTAAAATTCATTTTGAGGC AT-3′) and wsv024 probe (5′-Cy3-GATTTATCAAGTACGAAAAGGATAT-3′). After washing three times with PBS, the hemocytes were labeled with DAPI (4′, 6-diamidino-2-phenylindole) (50 ng/ml) (Sigma, USA) for 5 min. The images were captured using a CarlZeiss LSM710 system (Carl Zeiss, Germany). Fluorescence *in situ* hybridization assay was biologically repeated for three times.

### Statistical Analysis

All assays were carried out in three biological replicates and the data were presented as mean ± standard deviation. Data were analyzed by one-way analysis of variance (ANOVA) to obtain the mean values and standard deviation of biological repeats.

## Results

### Involvement of Host miR-1000 in Virus Infection

**Figure 1:**
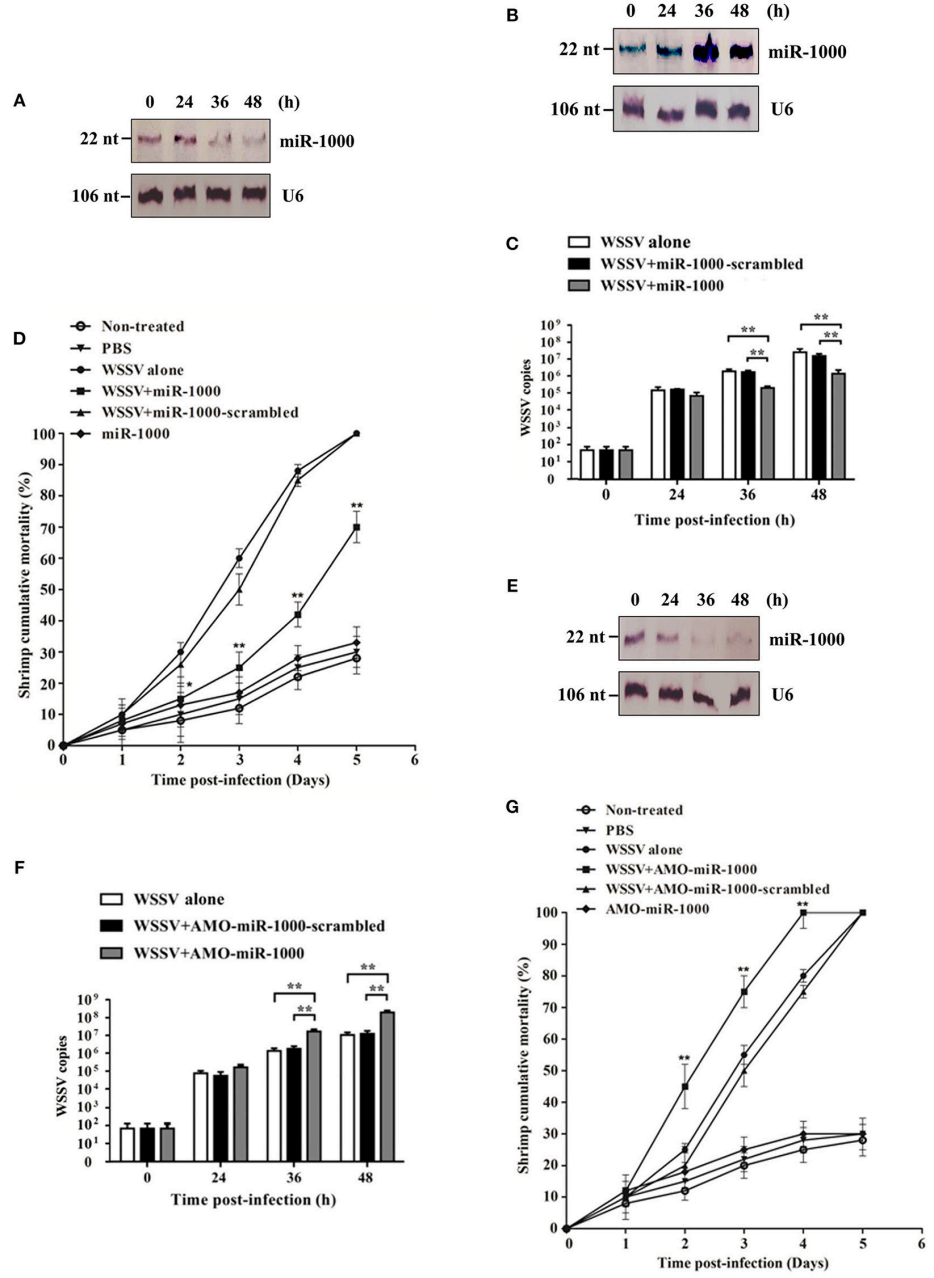
Involvement of host miR-1000 in virus infection. **(A)** Downregulation of miR-1000 in shrimp in response to virus infection. Shrimp were challenged with WSSV and then subjected to Northern blot analysis to detect miR-1000 expression level. U6 was used as a control. Numbers indicated the time points post-infection. **(B)** Overexpression of miR-1000 in shrimp. Shrimp were injected with miR-1000 and WSSV. At different time points post-infection, the expression level of miR-1000 was evaluated using Northern blot. **(C)** Effects of miR-1000 overexpression on WSSV infection. Shrimp were co-injected with miR-1000 or miR-1000-scrambled and WSSV, followed by the detection of WSSV copies. WSSV alone was used as a positive control. **(D)** Cumulative mortality of shrimp. PBS alone, miR-1000 alone and non-treated shrimp were used as negative controls. **(E)** Silencing of miR-1000 in shrimp. Shrimp were co-injected with AMO-miR-1000 and WSSV. At different time points post-infection, the expression level of miR-1000 was evaluated using Northern blot. **(F)** Influence of miR-1000 silencing on virus infection. **(G)** Cumulative mortality of shrimp. As negative controls, non-treated shrimp and shrimp injected with PBS or miR-1000 were assayed. Data presented here were the mean ± standard deviation of three independent experiments. Significant differences are indicated by asterisks (^*^p < 0.05; ^**^p < 0.01).

To explore the role and the relevant mechanism of miR-1000 during virus infection of shrimp, shrimp were challenged with WSSV, followed by the content detection of miR-1000. As in our previously study (10), miR-1000 was downregulated in shrimp in response to WSSV infection, indicating its involvement in virus infection (Figure 1A).

To further assess the relationship between miR-1000 and virus infection, miR-1000 was overexpressed and/or silenced in shrimp, followed by evaluation of the virus infection. The results indicated that when miR-1000 was overexpressed in shrimp (Figure 1B), the WSSV copies and the shrimp mortality were significantly decreased compared with the controls (Figures 1C,D), suggesting that miR-1000 played a negative role during virus infection. On the other hand, when miR-1000 was silenced in shrimp (Figure 1E), the WSSV copies and shrimp mortalities were significantly increased in comparison with the controls (Figures 1F,G).

All these data indicates that miR-1000 could reduce WSSV infection in shrimp, suggesting its antiviral activity *in vivo*.

### Interaction Between Host miR-1000 and Its Target Viral Genes

**Figure 2:**
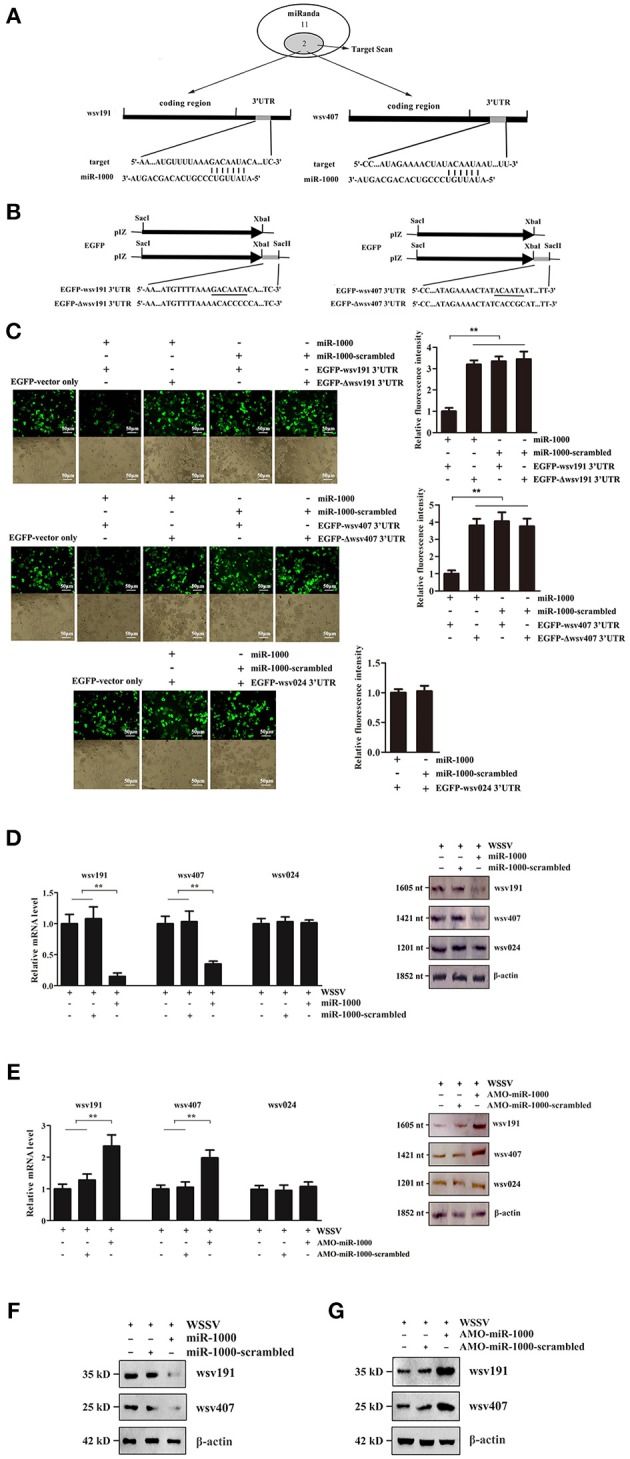
Interaction between host miR-1000 and its target viral genes. **(A)** Prediction of WSSV genes targeted by miR-1000. Numbers indicated the number of potential genes predicted by the algorithms. **(B)** Construction of the wild-type and mutated 3′UTRs of *wsv191* and *wsv407* genes. The sequences targeted by miR-1000 were underlined. **(C)** Direct interactions of miR-1000 with *wsv191* and *wsv407* genes in insect cells. High Five cells were co-transfected with miR-1000 and different plasmids. As controls, miR-1000-scrambled, EGFP-Δwsv191-3′UTR and EGFP-Δwsv407-3′UTR were included in the transfection. The interaction between miR-1000 and wsv024, a non-target gene of miR-1000, was also evaluated. At 48 h after co-transfection, the fluorescence intensities of the cells were examined (up). The numbers of cells were indicated with bright-field microscopy (down). Scale bar, 50 μm. **(D)** Influence of miR-1000 overexpression on the *wsv191* or *wsv407* mRNA level *in vivo*. MiR-1000 or miR-1000-scrambled was injected into WSSV-infected shrimp for 48 h, then the level of *wsv191* and *wsv407* mRNA was determined using quantitative real-time PCR (up) and Northern blot analysis (down). WSSV alone was used as a control. **(E)** Effects of miR-1000 silencing on the *wsv191* and *wsv407* mRNA level *in vivo*. At 48 h after treatment, the level of *wsv191, wsv407*, and *wsv024* mRNA in shrimp was determined using quantitative real-time PCR (up) and Northern blot analysis (down). **(F)** Impact of miR-1000 overexpression on the wsv191 and wsv407 protein level *in vivo*. Western blot analysis was carried out to evaluate the viral wsv191 and wsv407 protein levels. β-actin was used as a control. **(G)** Effects of miR-1000 silencing on the wsv191 and wsv407 protein levels *in vivo*. Data represented three independent experiments (^**^p < 0.01).

To find out the antiviral mechanism of host miR-1000, the virus genes targeted by miR-1000 were predicted using bioinformatics. The results showed that only two WSSV genes (*wsv191* and *wsv407*) might be the target genes of miR-1000 (Figure 2A).

To characterize the interaction between miR-1000 and *wsv191* or *wsv407*, the 3′UTR of *wsv191* and *wsv407* were cloned into the pIZ/V5-His vector, generating pIZ/EGFP-wsv191-3′UTR and pIZ/EGFP-wsv407-3′UTR (Figure 2B). As a control, the interaction between miR-1000 and wsv024, a non-target gene of miR-1000, was evaluated. The results showed that the fluorescence intensities of the insect High Five cells transfected with the empty EGFP vector, miR-1000-scrambled and miR-1000+EGFP-Δwsv191-3′UTR or miR-1000+EGFP-Δwsv407-3′UTR were consistent (Figure 2C). On the other hand, the fluorescence intensities of insect cells co-transfected with miR-1000 and EGFP-wsv191-3′UTR or EGFP-wsv407-3′UTR were notably decreased compared with the controls (Figure 2C). There was however no interaction between miR-1000 and wsv024 (Figure 2C). These results showed that miR-1000 could directly target *wsv191* and *wsv407* genes.

To explore the interaction between miR-1000 and *wsv191* or *wsv407 in vivo*, the expression of miR-1000 was silenced or overexpressed in shrimp, followed by the detection of *wsv191* mRNA and *wsv407* mRNA. As a control, *wsv024*, a viral gene that was not targeted by miR-1000, was included in the analysis. The results showed that miR-1000 overexpression led to a significant decrease in the mRNA levels of *wsv191* and *wsv407* compared with the controls (Figure 2D), while the mRNA level of *wsv191* and *wsv407* was remarkably upregulated when miR-1000 was silenced (Figure 2E). On the other hand, the mRNA level of the non-target gene *wsv024* was not influenced by miR-1000 (Figures 2D,E). Similarly, Western blot analyses yielded similar results (Figures 2F,G). These data thus indicate that miR-1000 interacted with the viral genes *in vivo*, suggesting that miR-1000 could target the mRNAs of the viral genes *wsv191* and *wsv407*.

### Effects of *wsv191* and *wsv407* Gene Silencing on WSSV Infected Shrimps

**Figure 3:**
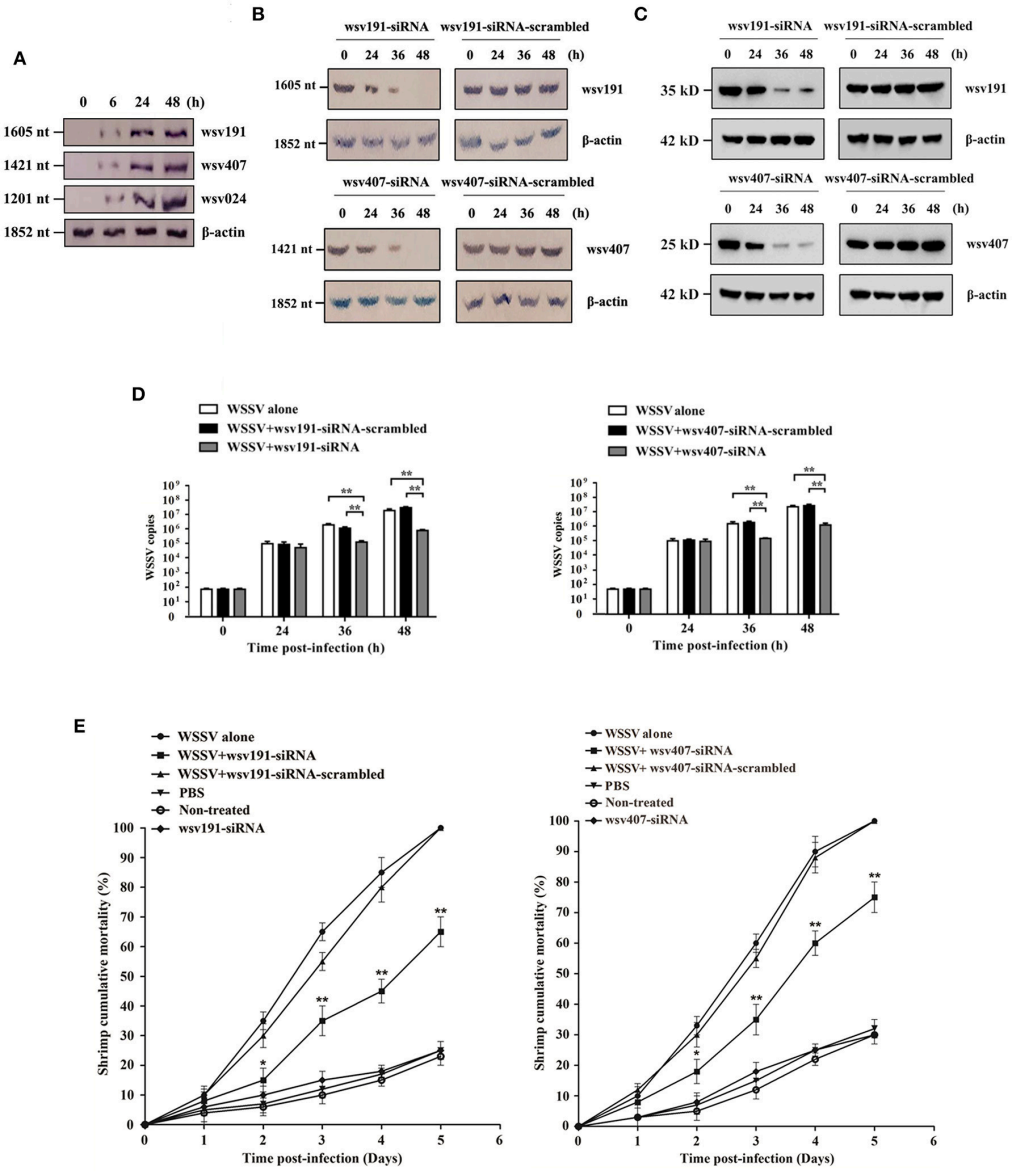
Effects of *wsv191* and *wsv407* gene silencing on WSSV infection. **(A)** Northern blot analysis of *wsv191, wsv407*, and *wsv024* gene expressions in WSSV-infected shrimp. Numbers indicated the time post-infection. **(B)** Silencing of *wsv191* and *wsv407* gene expressions in shrimp. Shrimp were infected with WSSV. At 48 h post-infection, shrimp were injected with wsv191-siRNA or wsv407-siRNA. Twelve hours later, shrimp were re-injected with the siRNA. At different time points after the last siRNA injection, the mRNA levels of wsv191 and wsv407 were determined using Northern blot. Numbers represented the time after the last siRNA injection of WSSV-infected shrimp. **(C)** Western blots showing the silencing of *wsv191* and *wsv407* in shrimp *in vivo*. **(D)** Influence of *wsv191* and *wsv407* gene silencing on WSSV virus infection in shrimp. Shrimp hemocytes were subjected to quantitative real-time PCR to determine WSSV copies. **(E)** Effects of *wsv191* and *wsv407* silencing on shrimp mortality. The mortality of shrimp per different treatment groups was examined daily. The treatments were shown on the top. Significant statistical differences between treatments were indicated with asterisks (^*^p < 0.05; ^**^p < 0.01).

In order to characterize the roles of wsv191 and wsv407 in WSSV infection, shrimp were infected with WSSV, followed by detection of *wsv191* mRNA and *wsv407* mRNA levels. The data showed that the mRNA levels of *wsv191* and *wsv407*, as well as the non-target miR-1000 gene *wsv024*, were increased in shrimp during WSSV infection (Figure 3A). As previously described (23), wsv191 encodes a non-specific nuclease, while wsv407 encodes a protein with no homology to any known proteins or motifs.

To examine the roles of wsv191 and wsv407 in virus infection, the expression of wsv191 and wsv407 was silenced in WSSV-infected shrimp. Northern blot (Figure 3B) and Western blot analysis (Figure 3C) showed that silencing of the expression of *wsv191* and *wsv407* by the sequence-specific siRNAs was attained at 36 and 48 h post-siRNA injection in WSSV-infected shrimp. Silencing of wsv191 and wsv407 significantly reduced WSSV copies in shrimp compared with the controls (Figure 3D). At the same, knockdown of *wsv191* and/or *wsv407* significantly decreased the mortality of WSSV-infected shrimp (Figure 3E).

Taken together, these results demonstrated that *wsv191* and *wsv407* played positive roles in WSSV infection of shrimp.

### Suppression of WSSV Infection by miR-1000 Via Targeting *wsv191* and *wsv407 in vivo*

**Figure 4:**
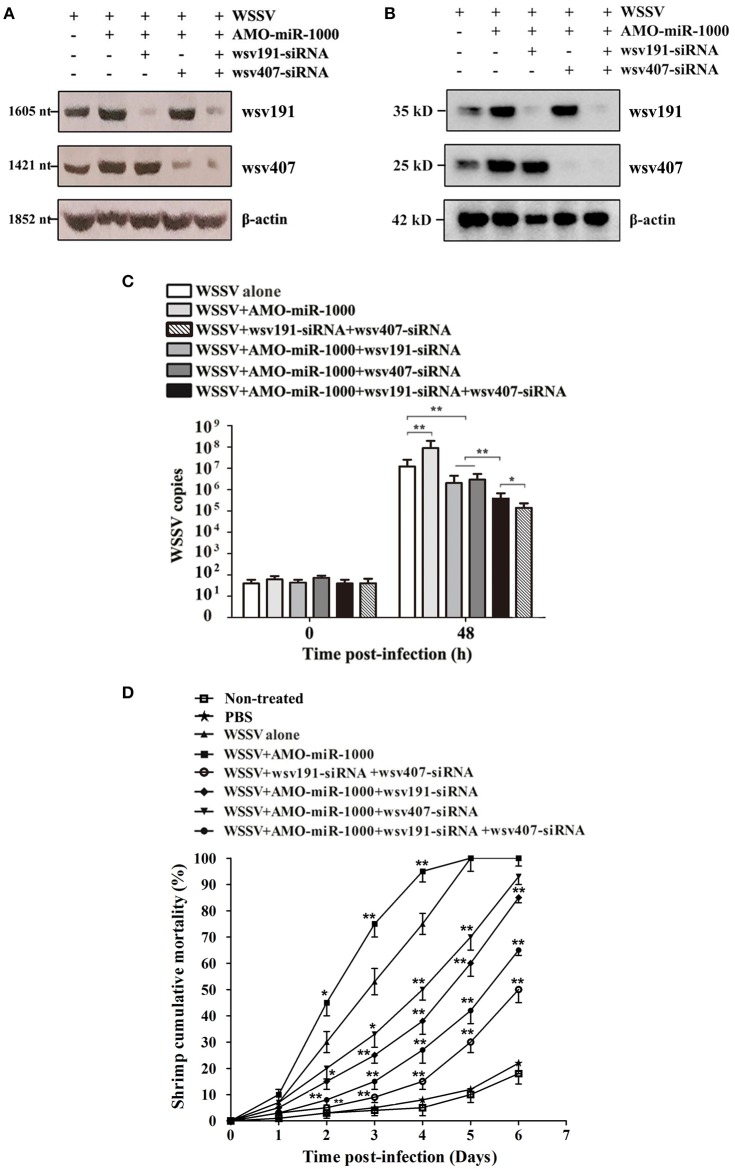
Suppression of WSSV infection by miR-1000 via targeting *wsv191* and *wsv407 in vivo*. **(A)** Northern blot analysis of *wsv191* and *wsv407* mRNA levels in shrimp. Shrimp were injected with WSSV, AMO-miR-1000, wsv191-siRNA and/or wsv407-siRNA. Forty 8 h later, shrimp hemocytes were subjected to Northern blot. **(B)** Western blot analysis of wsv191 and wsv407 protein levels. At 48 h after injection of WSSV, AMO-miR-1000, wsv191-siRNA, and/or wsv407-siRNA, shrimp hemocytes were analyzed with Western blot analysis to detect the wsv191 and wsv407 proteins. **(C)** Influence of miR-1000 silencing, wsv191 silencing or/and wsv407 silencing on WSSV infection in shrimp. The WSSV copy number of shrimp with different treatments was evaluated using quantitative real-time PCR. Numbers indicated the time points post-infection (^**^p < 0.01). **(D)** Cumulative mortality of shrimp. The mortality of shrimp was examined daily (^*^p < 0.05; ^**^p < 0.01).

To explore whether miR-1000 could suppress WSSV infection by targeting the viral genes *wsv191* and *wsv407* in shrimp, the effects of miR-1000, wsv191, and/or wsv407 silencing on virus infection were analyzed. Northern blot results showed that the mRNA levels of *wsv191* and *wsv407* were upregulated in shrimp injected with AMO-miR-1000 compared with the control (WSSV alone), but was downregulated in shrimp co-injected with AMO-miR-1000 and wsv191-siRNA or wsv407-siRNA (Figure 4A). Western blot analysis yielded similar results (Figure 4B), showing that the viral genes *wsv191* and *wsv407* were targeted by miR-1000 *in vivo*.

Analysis of WSSV copies and shrimp mortality revealed that virus infection was enhanced when miR-1000 was silenced, but was significantly diminished when shrimp were co-treated with miR-1000 and wsv191-siRNA or/and wsv407-siRNA (Figures 4C,D). This observation suggests that miR-1000 suppressed virus infection by targeting *wsv191* and *wsv407* genes *in vivo*. Similarly, WSSV infection was significantly inhibited when shrimp were treated with WSSV+AMO-miR-1000+wsv191-siRNA+wsv407-siRNA and WSSV+wsv191 -siRNA+wsv407-siRNA (Figures 4C,D). However, the inhibitory effect of WSSV infection by treatment of shrimp with WSSV+wsv191-siRNA+wsv407-siRNA was more significant than treatment with WSSV+AMO-miR-1000+wsv191-siRNA +wsv407-siRNA (Figures 4C,D).

All these results indicate that miR-1000 could suppress WSSV infection in shrimp by targeting the *wsv191* and *wsv407* genes, which play positive roles in virus infection.

### Underlying Mechanism of the miR-1000-Mediated Simultaneous Targeting of *wsv191* and *wsv407* mRNAs

**Figure 5:**
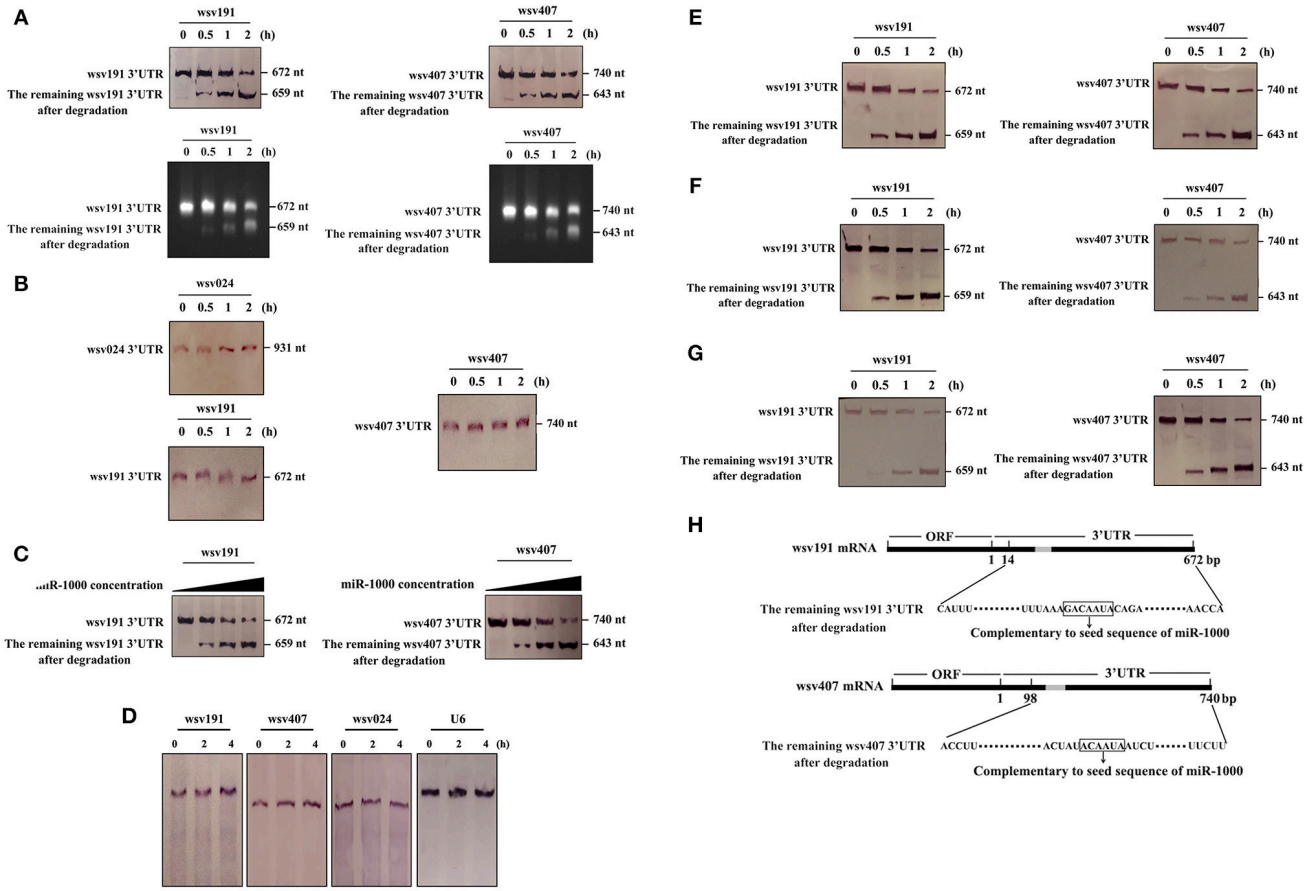
Underlying mechanism of the miR-1000-mediated simultaneous targeting of *wsv191* and *wsv407* mRNAs. **(A)** MiR-1000-mediated time-course degradation of *wsv191* and *wsv407* mRNAs. The 3′UTR of *wsv191* mRNA or *wsv407* mRNA and miR-1000 were incubated with shrimp Ago1 complex for different time intervals. The level of mRNA degradation was examined using Northern blot with *wsv191-*specific or *wsv407*-specific probes (up) and using agarose gel electrophoresis (down). Numbers indicated the incubation time. **(B)** Specificity of miR-1000-mediated degradation of target mRNAs. The 3′UTR of non-target gene *wsv024* mRNA and miR-1000, as well as the *wsv191* or *wsv407* mRNA 3′UTR and miR-1000-scrambled, were incubated with shrimp Ago1 complex for different time intervals, followed by Northern blot with *wsv024*-specific probe (up), *wsv191-*specific probe or *wsv407*-specific probe (down). Numbers showed the incubation time. **(C)** MiR-1000-concentration dependent degradation of *wsv191* mRNA or *wsv407* mRNA. The 3′UTR of *wsv191* mRNA or *wsv407* mRNA and shrimp Ago1 complex were incubated with miR-1000 at different concentrations for 2 h. Then the mixture was subjected to Northern blot. **(D)** Viral mRNA stability analysis during WSSV infection. Shrimp were injected with WSSV. Forty eight h later, the WSSV-infected shrimp were treated with actinomycin D. At different time points after treatment, shrimp were subjected to Northern blotting to detect *wsv191, wsv407*, and *wsv024* mRNAs. **(E)** MiR-1000-mediated synchronous degradation of *wsv191* mRNA and *wsv407*mRNA. The 3′UTRs of *wsv191* mRNA and *wsv407* mRNA as well as miR-1000 were simultaneously incubated with shrimp Ago1 complex for different time intervals. Subsequently, the mixture was analyzed by Northern blot. **(F,G)** Competitive degradation assays of *wsv191* and *wsv407* mRNA 3′UTRs mediated by miR-1000. To assess the competitive degradation between *wsv191* and *wsv407* mRNA 3′UTRs, 10-fold concentration of *wsv191* mRNA 3′UTR and 1-fold concentration of *wsv407* mRNA 3′UTR **(F)** or 1-fold concentration of *wsv191* mRNA 3′UTR and 10-fold concentration of *wsv407* mRNA 3′UTR **(G)** were mixed and then incubated with miR-1000 together with shrimp Ago1 complex for different time intervals. The mixture was analyzed by Northern blot. **(H)** Sequencing of degraded 3′UTRs of *wsv191* mRNA and *wsv407* mRNA. The arrows indicated the sequences of *wsv191* mRNA 3′UTR and *wsv407* mRNA 3′UTR complementary to the seed sequence of miR-1000.

To investigate the mechanism of miR-1000-mediated suppressions of multiple target gene expressions, the degradation of target mRNAs by miR-1000 was examined using Northern blot and agarose gel electrophoresis as described in our previous study (19). The data showed that the amount of degraded mRNA 3′UTR mediated by miR-1000 was gradually increased time-dependently (Figure 5A), indicating that miR-1000 could trigger the mRNA degradation of *wsv191* or *wsv407*. At the same time, agarose gel electrophoresis results showed that the miR-1000-mediated degradation of target mRNAs generated a specific band, the amount of which was gradually increased with incubation time (Figure 5A). To determine the specificity of the miR-1000-mediated mRNA degradation of target genes, the 3′UTR of *wsv191* mRNA and *wsv407* mRNA was incubated with miR-1000-scrambled and shrimp Ago1 complex. The results showed that there was no degraded band (Figure 5B). On the other hand, when miR-1000 and shrimp Ago1 complex were incubated with the mRNA 3′UTR of non-target *wsv024* gene, which was predicted to be a potential target of miR-1000 by the software MiRanda, Pictar, and miRInspector, no degraded band was observed (Figure 5B). These data therefore indicate that the miR-1000-mediated mRNA degradation of target genes was specific. Furthermore when the 3′UTR of *wsv191* mRNA and *wsv407* mRNA was incubated with miR-1000 at different concentrations, the degraded level of *wsv191* mRNA 3′UTR or *wsv407* mRNA 3′UTR was increased with increasing concentration of miR-1000 (Figure 5C), indicating that the miR-1000-mediated target mRNA degradation was miRNA-concentration dependent. To examine the stability of *wsv191, wsv407*, and *wsv024* mRNAs, actinomycin D was injected into WSSV-infected shrimp. The results showed that the *wsv191, wsv407*, and *wsv024* mRNAs were stable (Figure 5D). These data indicated that miR-1000 could mediate the mRNA degradation of its targets.

To explore whether there was a simultaneously mRNA degradation of *wsv191* and *wsv407*, miR-1000 was incubated with the 3′UTR of *wsv191* mRNA, *wsv407* mRNA and shrimp Ago1 complex. The results revealed that the degraded levels of *wsv191* and *wsv407* mRNAs increased time-dependently, suggesting that miR-1000 could mediate the simultaneous mRNA degradation of *wsv191* and *wsv407* (Figure 5E). Next, to examine whether there was competitive degradation between *wsv191* and *wsv407* mRNA 3′UTRs mediated by miR-1000, the *wsv191* mRNA 3′UTR at 10-fold concentration and that of *wsv407* mRNA 3′UTR at 1-fold concentration were incubated together with miR-1000 and shrimp Ago1 complex for different time intervals. The results indicated degradation of *wsv191* and *wsv407* mRNA 3′UTRs were not affected by different concentrations of mRNAs (Figure 5F). Similar results were obtained when 1-fold concentration of *wsv191* mRNA 3′UTR and 10-fold concentration of *wsv407* mRNA 3′UTR were mixed and incubated with miR-1000 and shrimp Ago1 complex (Figure 5G). All these data indicate that under our experimental conditions there was no competition between the two viral genes for miR-1000 mediated silencing.

To confirm the degradation of target mRNAs by miR-1000, the remaining 3′UTRs of *wsv191* mRNA and *wsv407* mRNA after miR-1000-mediated degradation were sequenced. The data showed that miR-1000 could mediate the degradation of the 3′UTRs of *wsv191* mRNA and *wsv407* mRNA, generating degraded fragments containing sequence complementary to the seed sequence of miR-1000 (Figure 5H).

All these data put together revealed that miR-1000 could trigger the mRNA degradation of multiple targets and that the miRNA-mediated mRNA degradation stopped near the mRNA 3′UTR sequence complementary to the seed sequence of miR-1000.

### Co-localization of miR-1000 and Its Target mRNAs in Shrimp *in vivo*

**Figure 6:**
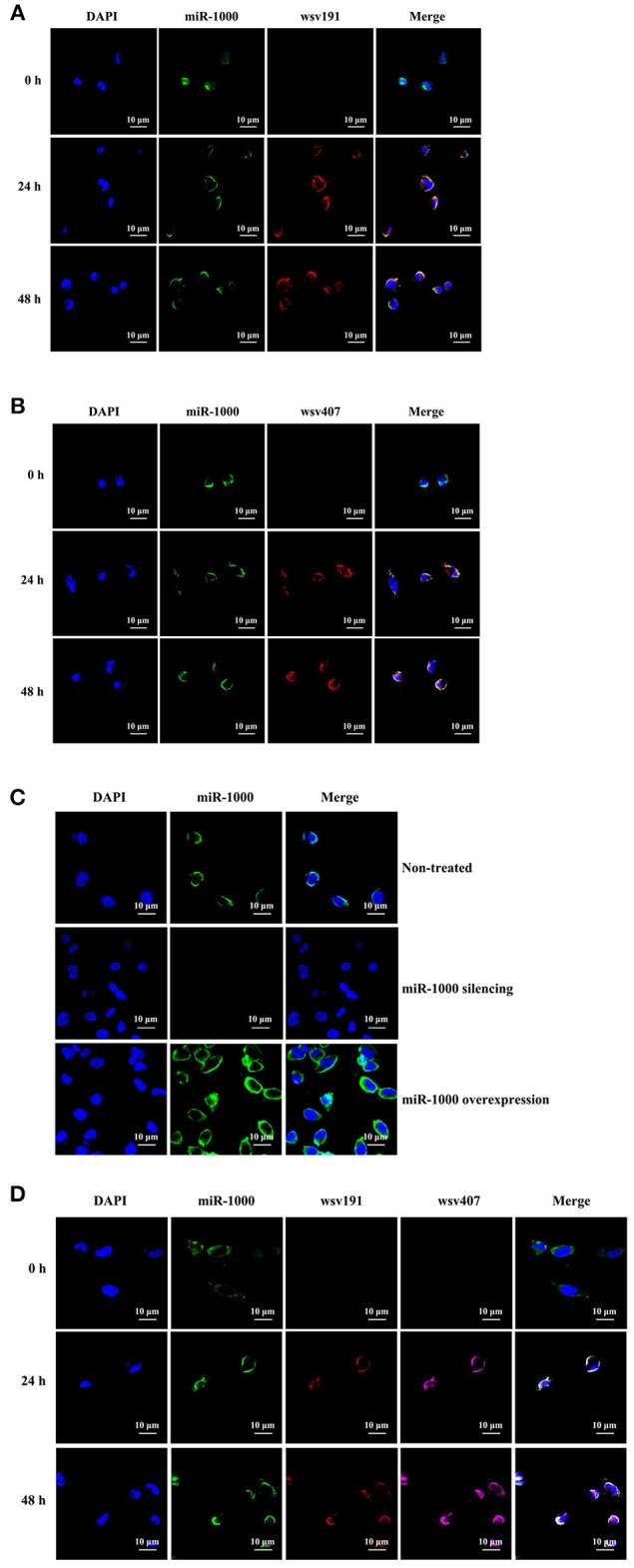
Co-localization of miR-1000 and its target mRNAs in shrimp *in vivo***. (A)** Co-localization of miR-1000 and *wsv191* mRNA in shrimp hemocytes. Shrimp were infected with WSSV. At different time points post-infection, miR-1000, *wsv191* mRNA, and nuclei of hemocytes were respectively detected with FAM-labeled miR-1000 probe (green), Cy3-labeled wsv191 probe (red), and DAPI (blue). Scale bar, 10 μm. **(B)** Co-localization of miR-1000 and *wsv407* mRNA in shrimp hemocytes. Shrimp hemocytes were examined by FAM-labeled miR-1000 probe (green) and Cy5-labeled wsv407 probe (red). Scale bar, 10 μm. **(C)** Specificity of miR-1000 probe. Shrimp were injected with AMO-miR-1000 or miR-1000 mimic. At 48 h after injection, miR-1000 was detected with FAM-labeled miR-1000 probe (green). Scale bar, 10 μm. **(D)** Evaluation of co-localization of miR-1000, *wsv191* mRNA, and *wsv407* mRNA in shrimp hemocytes. The WSSV-infected shrimp hemocytes were subjected to fluorescence *in situ* hybridization with FAM-labeled miR-1000 probe (green), Cy3-labeled wsv191 probe (red), and Cy5-labeled wsv407 probe (pink), respectively. Scale bar, 10 μm.

To investigate the targeting of *wsv191* and *wsv407* mRNAs by miR-1000 *in vivo*, the co-localization of miR-1000 and its target mRNAs in shrimp hemocytes was examined. The results of fluorescence *in situ* hybridization assays revealed that miR-1000 did co-localize with *wsv191* mRNA and *wsv407* mRNA in hemocytes of WSSV-infected shrimp (Figures 6A,B). To evaluate the specificity of the probe used, shrimp hemocytes overexpressing miR-1000 or depleted of miR-1000 was analyzed. The data showed that miR-1000 could not be detected in hemocytes of miR-1000-silenced shrimp (Figure 6C), indicating the miR-1000 probe was specific. These findings demonstrated that miR-1000 specifically interacted with its targets *in vivo*.

When miR-1000, *wsv191* mRNA, and *wsv407* mRNA were labeled simultaneously, it was found that miR-1000, *wsv191* mRNA, and *wsv407* mRNA were co-localized in WSSV-infected shrimp hemocytes (Figure 6D). These data further revealed that miR-1000 could simultaneously target *wsv191* and *wsv407* mRNAs *in vivo*.

Based on the above findings, it could be concluded that host miR-1000 suppressed virus infection by simultaneously targeting multiple viral genes *in vivo*.

## Discussion

The innate immune system of invertebrates provides the immediate defense against virus infection, which mainly depends on host-virus interactions (1). During virus infection, the virus genome is transported into the host nucleus to produce viral mRNAs using host transcriptional machinery (24). Viruses can encode a number of viral proteins that impact on host antiviral responses, thereby enabling viral replication via diverse mechanisms. Mimivirus, a DNA virus from the genus *Acanthamoeba*, is a parasite of multiple species, and encodes more than 900 viral genes (25). The human immunodeficiency virus HIV-1, is a retrovirus, which establishes persistent infection and leads to acquired immunodeficiency syndrome. HIV-1 is reported to encode about 15 genes during the infection of target cells (26). In WSSV, the viral genome has the capacity to encode more than 180 viral genes during the virus invasion process (27). There are therefore many viral genes involved in virus-host interactions. Thus, how the host is able to immediately and effectively regulate the expressions of all these viral genes needs to be explored. It is well known that miRNAs, a large category of small non-coding RNAs, downregulate the expressions of their target genes (12). In our previous studies, high-throughput sequencing of shrimp miRNAs identified 31 host miRNAs associated with virus infection, 25 and 6 of which were, respectively, upregulated and downregulated in shrimp in response to WSSV challenge (4, 6). Among the miRNAs associated with virus infection, some miRNAs (miR-1, miR-7 and miR-34) were highly conserved in animals, and had functions in the same pathways in shrimp, fruit fly and humans through targeting the same or similar genes (4). Host miRNAs can play essential roles in host antiviral immunity to fight against virus invasion (19), but some virus genes have evolved short-length 3′UTRs (28, 29). These virus genes with short-length 3′UTRs can attenuate the effects of host miRNAs (30, 31). Generally, an individual miRNA possesses multiple target genes (32), implying that the host miRNAs can be the most efficient regulators of virus gene expressions. However, the role of multi-gene regulation by miRNA in virus infection is largely unknown. In this study, the findings revealed that a host miRNA could simultaneously target multiple virus genes (*wsv191* and *wsv407*) and synchronously trigger the mRNA degradation of its targets *in vivo*, leading to the inhibition of virus infection. The results of the present investigation indicated that miR-1000 silencing led to significant increases in WSSV copies and shrimp mortality compared with shrimp injected with only WSSV, while our previous study revealed that the mortality of miR-1000-silencd shrimp was slightly increased (10). This discrepancy could be due to variations in the virus inoculum used in the two studies. To ensure that increased copies of WSSV could be detectable, a lower concentration of WSSV inoculum (10^4^ copies/shrimp) was used to trigger virus infection of shrimp in the present study. Our findings demonstrated that *wsv191* and *wsv407* had positive effects on virus infection in shrimp. Given that *wsv191* encodes a non-specific nuclease, which can hydrolyze to both DNA and RNA (23), it has previously been observed that double-stranded RNA treatment of *wsv191* significantly decreases the mortality of WSSV-infected *Litopenaeus vannamei* (33), which is consistent with our findings in this study. In herpes simplex virus, nuclease is required for efficient processing of viral DNA replication intermediates (34), an observation which is similar to our present data, that seems to suggest that nucleases play an important role in virus infection. While the functions of *wsv407* are still not clear, sequence analysis showed that the most homologous proteins of wsv407 were E3 ligase and wsv249 [an ubiquitin E3 ligase (35)] of WSSV. Some studies have shown that the virus-encoded E3 ligases play positive roles during virus infection (36, 37). Therefore, our study revealed some possible role of wsv407 in WSSV infection.

Our previous investigation indicated that miR-1000, a conserved miRNA in invertebrates, could target the *p53* gene of shrimp to suppress apoptosis and WSSV infection (10). In this context, it appears shrimp miR-1000 took had effects on shrimp antiviral immunity by targeting host gene (*p53*) and virus genes (*wsv191* and *wsv407*). As shown in Figure 1A, the expression level of miR-1000 was decreased in shrimp during WSSV infection, suggesting that the virus could suppress the expression of host antiviral miRNA. To escape from host immune responses, viruses can suppress the expressions of host antiviral genes (38). Therefore, our study provided novel knowledge about the miRNA-mediated regulatory mechanism of gene expression during virus-host interactions.

RNA interference (RNAi) mediated by miRNAs can function in antiviral immune responses via post-transcriptional regulation of gene expression (39). By binding to the 3′UTRs of target transcripts, a miRNA mediates translational repression or direct mRNA degradation to suppress the expressions of the target genes (12). When a miRNA sequence is fully complementary to its target mRNA, the miRNA is capable of inducing endonucleolytic cleavage of its target mRNA at the middle of the matching region (40). Since the majority of animal miRNAs are not completely complementary to their target mRNAs, miRNAs often silence the target gene expression through mRNA degradation by 5′-3′ exonucleolytic digestion in animals (41–43). During miRNA-mediated mRNA degradation process in animals, mRNAs are first deadenylated by the CAF1 (chromatin assembly factor 1)-CCR4 (C-C motif chemokine receptor 4)-NOT1 (CCR4-NOT transcription complex subunit 1) deadenylase complex, and then the deadenylated mRNAs are decapped by the enzyme DCP2 (decapping protein 2) (44). The decapped mRNAs are further degraded mainly by the cytoplasmic 5′-3′ exonuclease XRN1 (5′-3′ exoribonuclease 1) (44). A large number of studies have shown that mRNA degradation mainly contribute to the miRNA-mediated gene silencing in vertebrates (45, 46). In colorectal cancer cells, the expression of beta-transducin repeats-containing protein 1 (βTrCP1), a substrate recognition subunit for the SCF^β*TrCP*^ E3 ubiquitin ligase, is suppressed by miR-183 through direct mRNA degradation (47). In HCT116 cells, *TP53* mRNA is directly degraded by miR-125b, while the process is interrupted by poly(A)-specific ribonuclease (PARN) (48). In the present study, the findings demonstrated that the miR-1000-mediated 5′-3′ exonucleolytic digestion of target mRNA generated the degraded mRNA fragment which contained the sequence complementary to the seed sequence of miR-1000 in shrimp. Our study showed that a miRNA could mediate 5′-3′ exonucleolytic digestion of target mRNAs in invertebrate and this 5′-3′ exonucleolytic digestion stopped near the mRNA 3′UTR sequence complementary to the seed sequence of miRNA. Therefore, our findings contributed new insights into the miRNA-mediated degradation of target mRNAs in shrimp.

## Author Contributions

XZ and YG designed the research and conceived the study. YG and CJ performed the experiments. XZ and YG wrote the manuscript.

### Conflict of Interest Statement

The authors declare that the research was conducted in the absence of any commercial or financial relationships that could be construed as a potential conflict of interest.
